# Mutations in SIX1 Associated with Branchio-oto-Renal Syndrome (BOR) Differentially Affect Otic Expression of Putative Target Genes

**DOI:** 10.3390/jdb9030025

**Published:** 2021-06-30

**Authors:** Tanya Mehdizadeh, Himani D. Majumdar, Sarah Ahsan, Andre L. P. Tavares, Sally A. Moody

**Affiliations:** Department of Anatomy & Cell Biology, School of Medicine and Health Sciences, George Washington University, Washington, DC 20037, USA; tanya_414@gwmail.gwu.edu (T.M.); hmajumdar@gwu.edu (H.D.M.); sarahahsan@gwmail.gwu.edu (S.A.); tavaresa@gwu.edu (A.L.P.T.)

**Keywords:** neural crest, cranial placodes, transcription, *Xenopus*

## Abstract

Several single-nucleotide mutations in *SIX1* underlie branchio-otic/branchio-oto-renal (BOR) syndrome, but the clinical literature has not been able to correlate different variants with specific phenotypes. We previously assessed whether variants in either the cofactor binding domain (V17E, R110W) or the DNA binding domain (W122R, Y129C) might differentially affect early embryonic gene expression, and found that each variant had a different combination of effects on neural crest and placode gene expression. Since the otic vesicle gives rise to the inner ear, which is consistently affected in BOR, herein we focused on whether the variants differentially affected the otic expression of genes previously found to be likely Six1 targets. We found that V17E, which does not bind Eya cofactors, was as effective as wild-type Six1 in reducing most otic target genes, whereas R110W, W122R and Y129C, which bind Eya, were significantly less effective. Notably, V17E reduced the otic expression of *prdm1*, whereas R110W, W122R and Y129C expanded it. Since each mutant has defective transcriptional activity but differs in their ability to interact with Eya cofactors, we propose that altered cofactor interactions at the mutated sites differentially interfere with their ability to drive otic gene expression, and these differences may contribute to patient phenotype variability.

## 1. Introduction

Branchio-otic/Branchio-oto-renal (BOR) syndrome is an autosomal dominant developmental disorder responsible for a variable combination of developmental defects including hyoid fistulas/cysts, inner, middle and external ear malformations leading to conductive and/or sensorineural hearing loss, and in some cases kidney dysmorphology [1,2,3]. Point mutations that result in single amino acid substitutions have been identified in about half of BOR cases, including SIX1, a homeodomain-containing transcription factor (~4% of patients), and EYA1, a co-factor that binds to SIX1 to modify its transcriptional activity (~40% of patients) [3,4]. Like other members of the SIX family, SIX1 contains two highly conserved domains: a SIX-type homeodomain (HD) that binds to DNA, and an N-terminal SIX domain (SD) that binds to cofactor proteins that modify its transcriptional activity [5,6,7,8]. All of the reported *SIX1* mutations in BOR are located in either the SD or the HD [9,10,11,12,13,14]. Studies in several vertebrates indicate that *Six1* is a crucial regulator of cranial placode development [15,16,17,18,19]. Six1 loss-of-function studies demonstrate reduced expression of placode genes and/or defects in otic development [20,21,22,23,24,25,26,27,28,29,30,31,32,33]. Conversely, *Six1* gain-of-function studies show expansion of placode domains at the expense of the adjacent epidermal, neural crest and neural plate regions [21,29,34,35]. 

In BOR, there is considerable variability in the presence, type and severity of clinically relevant abnormalities, even among family members carrying the same mutation, and as a consequence it has not been possible to correlate the type of mutation with the clinical presentation [9,10,11,12,36,37]. To address whether some of the phenotype variability might be attributed to changes in gene expression in the embryonic precursors of the affected cranial tissues, we tested their effects in *Xenopus* embryos. Because the amino acid sequence of *Xenopus* Six1 in the SD and HD is 100% identical to that of human, we introduced four of the human mutations into *Xenopus Six1* [14]: two in the SD and two in the HD. The mutations include a V17E mutation in the first α-helix of the SD that interferes with the SIX1-EYA interaction and subsequent translocation of EYA to the nucleus, an R110W mutation in the sixth α-helix of the SD that decreases the EYA/SIX interaction and reduces transcriptional activation, a W122R mutation adjacent to the N-terminus of the HD that disturbs interactions with EYA1 and is transcriptionally deficient, and a Y129C mutation in the N-terminal region of the HD that significantly reduces DNA binding and transcriptional activity [10,12,13,14]. We found that expressing each Six1 mutant protein in a wild-type embryo had different effects on genes expressed by the precursors of the affected BOR tissues, i.e., the neural border, neural crest, and the preplacodal ectoderm, (PPE), as well as a few otic vesicle genes [14]. In addition, morphological assessment of the tadpole inner ear demonstrated that the auditory and vestibular structures formed, but the volume of the lumen, the otic capsule, otoconia and some sensory hair cell end-organs were differentially affected by the BOR variants [14]. 

Large-scale screens in flies and vertebrates have identified hundreds of potential Six1 transcriptional targets in numerous tissues and several developmental stages [38,39,40,41,42]. Therefore, we decided to assess whether the previous observations were the result of Six1 sitting at the apex of the cranial placode gene regulatory network, and thereby altering all downstream events, or the consequence of altering the expression of specific Six1 transcriptional targets. To accomplish this, we chose five putative targets based on the afore-mentioned screens (*eya2*, *prdm1*, *spry1*, *tspan13*, *zbtb16*), as well as one novel cofactor (*pa2g4*) [43]. *eya2* is expressed in the pre-placodal region and acts as a Six1 cofactor to promote PPE gene expression and suppress neural plate and neural crest cell fates [29,44,45]. *prdm1* (aka *blimp1*) encodes a transcription factor that plays a role in pharyngeal arch, neural crest and placode formation [46,47,48,49]. *spry1* encodes a negative regulator of FGF signaling and is expressed in several craniofacial tissues [50,51,52]. *tspan13*, a member of the *tetraspanin* gene family, encodes a cell-surface protein that mediates signal transduction events in developing systems and cancers [53]. *zbtb16* encodes a Krueppel C2H2-type zinc finger protein expressed in craniofacial and several other developing tissues [54,55]. *pa2g4* (aka *ebp1*) is expressed in craniofacial tissues and cancers [56,57,58], and its protein binds to Six1 to regulate the development of neural crest and PPE [43].

First, we used translation-blocking morpholino-mediated knockdown of endogenous Six1 protein to demonstrate that each of these genes required the presence of Six1 for expression in the otic vesicle (OV). By introducing activating or repressing versions of Six1 into embryos, we found that in the absence of exogenously supplied Eya cofactor each gene was repressed by wild-type Six1; for two OV genes, repression depended on the level of Six1. To assess whether the BOR variants alter otic gene expression in a manner similar to wild-type Six1, they were expressed in embryos containing the normal endogenous level of wild-type Six1 similar to BOR patients that express one normal allele and one mutant allele. We found that V17E, which does not bind to cytoplasmic Eya1 or translocate it into the nucleus [14], was as effective as wild-type Six1 in reducing four of the otic target genes. In contrast, R110W, W122R and Y129C, which bind Eya1 and translocate it into the nucleus [14], were significantly less effective. It was notable that whereas V17E reduced the otic expression of *prdm1* more than Six1WT, i.e., was more repressive, R110W, W122R and Y129C had the opposite effect by expanding it. Since these BOR variants each have defective transcriptional activity, but differ in their ability to interact with Eya1, we propose that altered cofactor interactions at the mutated sites differentially interfere with their ability to drive the otic expression of target genes, and these differences may contribute to patient phenotype variability.

## 2. Materials and Methods

### 2.1. Obtaining Embryos and Microinjections

Fertilized *Xenopus laevis* embryos were obtained by gonadotropin-induced natural mating of adult frogs [59]. Embryos were picked at the two-cell stage to accurately identify the dorsal and ventral animal blastomeres [59,60,61]. When these embryos reached the eight-cell stage, the dorsal-animal and ventral-animal blastomeres, which extensively give rise to the neural crest and cranial placodes [62], were microinjected on one side of the embryo as previously described [59], with 1nl of: (1) one of the *Six1* mRNAs (wild type, fusion protein, or mutant) mixed with β-galactosidase mRNA as a lineage tracer, or (2) a 1:1 mixture of two lissamine-tagged antisense morpholino oligonucleotides that previously were verified to effectively and specifically block the translation of endogenous Six1 (Six1-MOs) [21]. Following, embryos were cultured in a dilution series of Steinberg’s medium until fixation.

### 2.2. In Vitro Synthesis of mRNAs and Antisense RNA Probes

Transcripts encoding Six1 wild-type (Six1WT) [21], Six1WT SD+HD fused to the VP16 activation domain (Six1VP16) [21], Six1WT SD+HD fused to the En2 repressive domain (Six1EnR) [21], four different Six1 mutants (V17E, R110W, W122R, Y129C) [14] and a nuclear-localized βgalactosidase mRNA (as a lineage tracer) were synthesized in vitro according to manufacturer’s protocols (mMessage mMachine Kit, Thermo Fisher Scientific, Waltham, MA, USA). Plasmids encoding *eya2* (Open Biosystems, Huntsville, AL, USA), *prdm1* (Open Biosystems, Huntsville, AL, USA), *spry1* (Dharmacon, Lafayette, CO, USA), *tspan13* (Dharmacon, Lafayette, CO, USA), *zbtb16* (Source Bioscience, Nottingham, UK) and *pa2g4* (Open Biosystems, Huntsville, AL, USA) were purchased and sequenced in both directions to confirm identity. Digoxigenin-labeled antisense RNA probes for in situ hybridization (ISH) assays were synthesized from these plasmids in vitro according to manufacturer’s protocols (MEGAscript Kit; Thermo Fisher Scientific, Waltham, MA, USA).

### 2.3. Histochemistry, In Situ Hybridization (ISH) and Analyses

Embryos were cultured to otic pit (st 20–22) or otic vesicle (st 26–32) stages [63], fixed in 4% paraformaldehyde (in 0.1 M MOPS, 2 mM EGTA magnesium, 1 mM MgSO_4_, pH 7.4), stained for β-Gal histochemistry in those embryos injected with mRNA, and processed for ISH according to standard protocols [64]. The analysis included only those embryos in which either the β-Gal-positive (mRNA-injected) or lissamine-labeled (MO-injected) cells were located in the otic region, indicating that the injected reagent was targeted to the correct tissue. In each embryo, the intensity and size of the otic expression domain of each gene was compared between the injected, lineage-labeled side of the embryo to the uninjected, control side of the same embryo, providing paired analyses. Assays were repeated a minimum of three times on embryos derived from three different sets of outbred parents. At least two authors independently scored embryos for gene expression changes. Differences in the frequency of gene expression changes were tested for significance (*p* < 0.05) by the chi-square test (GraphPad Prism software, San Diego, CA, USA).

### 2.4. QPCR Analyses

Both cells of two-cell embryos were injected with either *Six1WT* mRNA (150 pg or 400 pg/cell) or mutant mRNAs (V17E, 150 pg; R110W 400 pg; W122R 400 pg; Y129C 400 pg/cell), grown to stage 32, and ten dissected heads were collected in TRI-reagent (Zymo Research, Tustin, CA, USA) and processed for RNA extraction with DNAse I treatment using the Direct-zol RNA Miniprep kit (Zymo Research, Tustin, CA, USA). cDNA was synthesized using the iScript Advanced cDNA Synthesis kit (Bio-Rad, Hercules, CA, USA). qPCR was performed using 5ng cDNA with the SsoAdvanced Universal SYBR Green Mix (Bio-Rad, Hercules, CA, USA). Primer sequences are listed in Table 1. qPCR of three biological replicates (ten heads per replicate) was performed in duplicate. PCR and data analysis were performed using a CFX Connect thermocycler (Bio-Rad, Hercules, CA, USA). Statistical analysis was performed with GraphPad Prism 9, with significance calculated by two-way ANOVA followed by Tukey’s multiple comparisons test.

## 3. Results

### 3.1. Expression of Several Otic Genes Requires Six1

**Figure 1:**
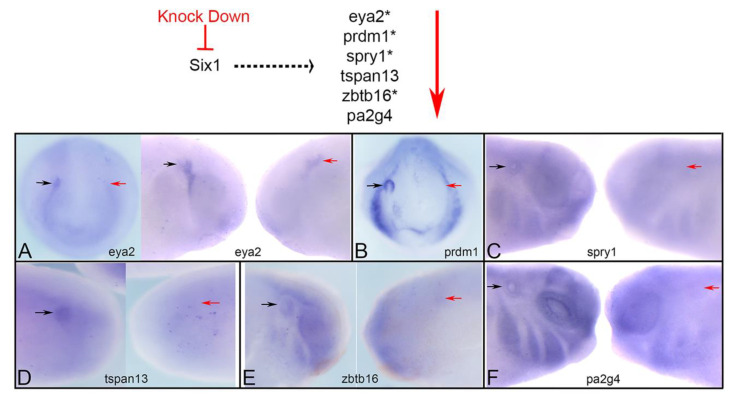
Six1 is required for otic gene expression. Top: Six1 is predicted to act upstream of the investigated genes. If correct, then the expression domain and/or intensity should be reduced (red arrow) when Six1 is knocked down. Those genes with an asterisk were shown to have Six1 bound to an enhancer in mouse E10.5 otic vesicles [42]. We found that the otic placode ((**A**), left image) or otic vesicle ((**A**), right image, (**B**–**F**)) expression of *eya2* (**A**), *prdm1* (**B**), *spry1* (**C**), *tspan13* (**D**), *zbtb16* (**E**) and *pa2g4* (**F**) was reduced on the MO-mediated knock-down side of each embryo (red arrow) compared to the control side (black arrow) of the same embryo. A, left image and B are frontal views; A, right image and C-F are side views. Dorsal is to the top.

Several microarray, RNAseq and ChIPseq assays have identified a large number of potential targets of Six1 transcriptional regulation [38,39,40,41,42]. We chose five putative targets from these lists based on their expression in the otic vesicle (OV), and one novel cofactor that interferes with Six1-Eya1 transcriptional activation [43]. To verify whether these genes are downstream of Six1 in the OV, we knocked down endogenous Six1 protein via injecting a previously validated mixture of two translation-blocking antisense morpholino oligonucleotides (Six1MOs) [21] into the neural crest/placode precursor blastomeres on one side of the embryo. If these genes require Six1, their expression domain and/or intensity would be reduced (Figure 1). We found that each gene was reduced in the OV of the majority of embryos (*eya2*, 83.1%, n = 124; *prdm1*, 96.7%, n = 60; *spry1*, 62.2%, n = 82; *tspan13*, 85.1%, n = 47; *zbtb16*, 72.1%, n = 61; *pa2g4*, 78.4%, n = 51; Figure 1). These results demonstrate that the OV expression of each gene is downstream of Six1 protein, which together with the previous, aforementioned screens, confirms that they are Six1 targets. 

**Figure 2:**
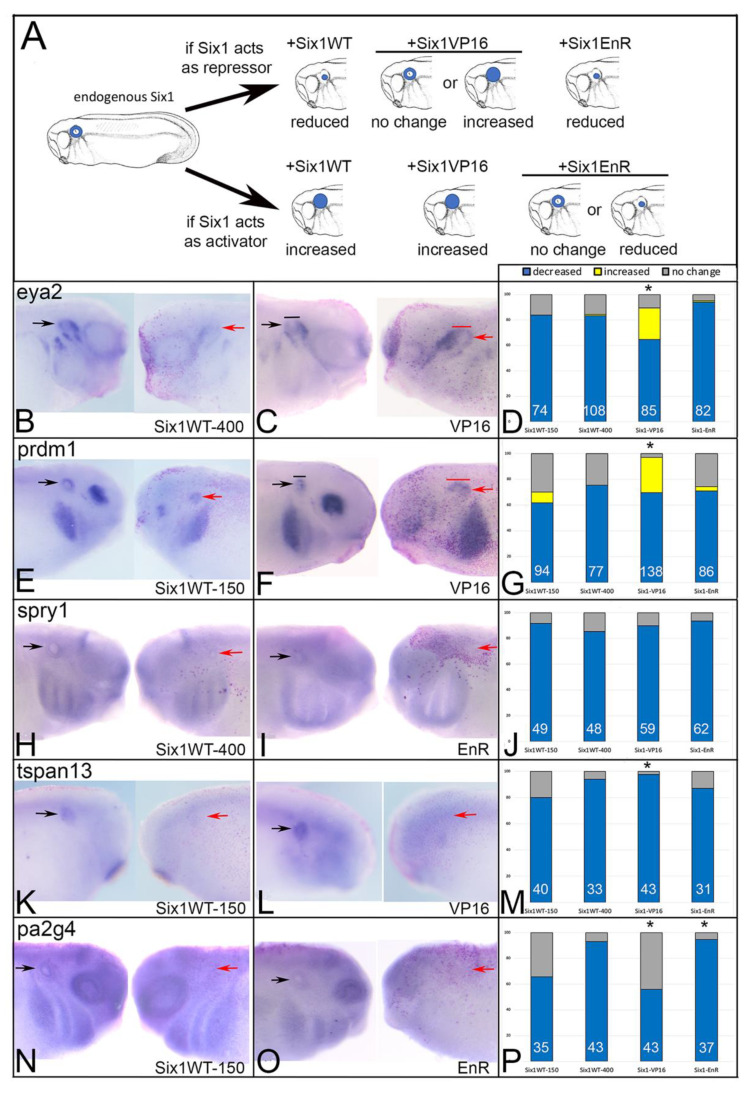
Increased Six1 alters otic gene expression. Transcripts encoding wild-type Six1 (Six1WT, either 150 or 400 pg), activating Six1 (VP16, 100 pg) or repressive Six1 (EnR, 100 pg) were microinjected into blastomeres that contribute to otic structures on one side of embryos containing endogenous levels of Six1. (**A**) The effects on otic vesicle gene expression (blue) were compared between embryos injected with *Six1WT* mRNA (+Six1WT) versus those injected with either *Six1VP16* (+Six1VP16) or *Six1EnR* (+Six1EnR) mRNAs. If Six1 acts as a repressor (top row), then additional Six1WT should reduce gene expression, Six1VP16 should either cause no change or increase it, and Six1EnR should also reduce it. If Six1 acts as an activator (bottom row), then additional Six1WT should increase gene expression, Six1VP16 should also increase it, and Six1EnR should cause either no change or reduce it. (**B**–**P**) Gene expression was assayed by ISH for *eya2* (**B**,**C**), *prdm1* (**E**,**F**), *spry1* (**H**,**I**), *tspan13* (**K**,**L**) and *pa2g4* (**N**,**O**). The control, uninjected side of each embryo is on the left and the mRNA-injected side of the same embryo is on the right. Otic gene expression on the control side is indicated by black arrows, and that on the mRNA-injected side by red arrows. In C and F, the width of the otic vesicle is indicated by a black (control) or red (injected) bar. Frequencies of the effects (blue, decreased expression; yellow, increased expression; grey, no change) are indicated by bar graphs (**D**,**G**,**J**,**M**,**P**). For *eya2* (**D**) and *prdm1* (**G**), the frequencies for Six1VP16 were significantly different from both Six1WT-150 and SixWT-400 (*, *p* < 0.0001). For *tspan13* (**M**), the frequencies for Six1VP16 were significantly different from Six1WT-150 (*, *p* < 0.05), but not from SixWT-400. For *pa2g4* (**P**), the frequencies for Six1VP16 were significantly different from SixWT-400 (*, *p* < 0.0001), and the frequencies for Six1EnR were significantly different from SixWT-150 (*, *p* < 0.01). White numbers inside each bar denotes the number of embryos analyzed.

Six1 acts as a transcriptional repressor except in the presence of Eya proteins [21,65,66,67]. To test whether target gene expression was either increased or decreased by supplying additional Six1 in the embryo we injected mRNA constructs that cause Six1 to be either activating (*Six1VP16*) or repressive (*Six1EnR*) in the absence of exogenously supplied Eya. Because these mRNAs synthesize protein that is in addition to endogenous levels, we compared their effects to those resulting from injecting *SixWT* mRNA (Figure 2A). If Six1 WT activates target gene transcription in the absence of exogenous Eya, one would expect the frequency at which a target gene changed to be indistinguishable from that of Six1VP16. Likewise, if Six1 WT represses target gene transcription, one would expect the frequency at which a target gene changed to be indistinguishable from that of Six1EnR.

We found that each putative target gene was reduced by Six1 via transcriptional repression. For *eya2*, Six1WT-150 (150 pg of mRNA), Six1WT-400 (400 pg of mRNA) and Six1EnR (100 pg of mRNA) reduced its expression at the same frequencies (*p* > 0.05), whereas Six1VP16 (100 pg of mRNA) caused a significantly lower frequency of repression and expanded expression in about 25% of the embryos (Figure 2B–D). Since the Six1VP16 phenotype frequencies were significantly different from Six1WT (*p* < 0.0001), whereas the Six1EnR frequencies were not, we posit that endogenous Six1 acts to repress *eya2* after its initial induction (which required earlier Six1) in the OV. Similar results were observed for *prdm1* (*p* < 0.00001; Figure 2E–G). For *spry1*, Six1VP16 and Six1EnR both repressed its expression at frequencies indistinguishable from either dose of Six1WT (Figure 2H–J), suggesting that its regulation by endogenous Six1 is more complicated; *spry1* may be both directly and indirectly regulated perhaps by involving different cofactors. For *tspan13*, Six1VP16 was significantly more repressive compared to Six1WT-150 pg (*p* < 0.05) but not different compared to Six1WT-400 pg. Six1EnR repressed *tspan13* at frequencies indistinguishable from either Six1WT dose (Figure 2K–M). These results suggest that different endogenous levels of Six1 may differentially regulate *tspan13* expression, as we previously observed for *sox2* in cranial ganglia formation [34]. Our results suggest that at high levels, Six1 reduces *tspan13* expression via transcriptional activation, whereas at low levels it reduces it via transcriptional repression. For *pa2g4*, Six1VP16 repressed its expression at a frequency indistinguishable from Six1WT-150, whereas Six1EnR repressed at a similar frequency as Six1WT-400 (Figure 2N–P). These results suggest that the endogenous levels of Six1 in the embryo differentially regulate *pa2g4* expression, but in contrast to *tspans13*, at high levels Six1 reduced *pa2g4* by transcriptional repression and at low levels reduced *pa2g4* via transcriptional activation. These experiments indicate that endogenous Six1 likely regulates these putative target genes by repression in the absence of Eya1, and for some this may depend on the level of protein. To determine whether the interactions are direct or involve other cofactors, a detailed assessment of the transcriptional complex components that sit on identified enhancers of each gene during OV formation will be an important pursuit.

### 3.2. BOR Mutations Differentially Reduce Cranial Expression of Putative Six1 Targets

**Figure 3:**
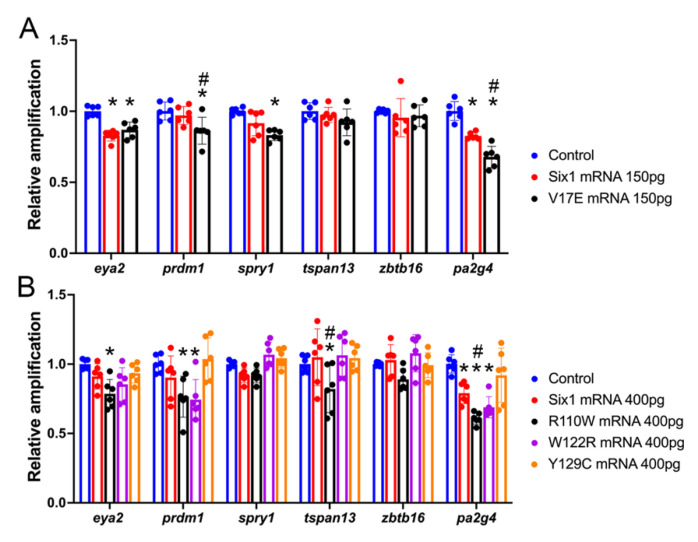
Gene expression changes in whole head samples assessed by qPCR. (**A**). Levels of expression in whole heads collected from uninjected, sibling-matched embryos (controls, blue bars), and embryos injected with mRNA encoding either Six1WT (red bars) or V17E (black bars). Significant differences from control (*p* < 0.05) are indicated by an asterisk; significant differences from Six1WT (*p* < 0.05) are indicated by #. (**B**). Levels of expression in whole heads collected from uninjected, sibling-matched embryos (controls, blue bars), and embryos injected with mRNA encoding either Six1WT (red bars), R110W (black bars), W122R (purple bars) or Y129C (orange bars). Significant differences from control (*p* < 0.05) are indicated by an asterisk. Significant differences from Six1WT (*p* < 0.05) are indicated by #. Data are from three independent samples run in duplicate.

To determine whether some of the *SIX1* mutations found in BOR patients affect the expression of the investigated otic genes, we performed qPCR analysis of mRNAs extracted from whole larval heads (Figure 3). In this approach, we compared the effect of an exogenously introduced BOR variant to that of exogenously introduced Six1WT to test whether the BOR variant had comparable activity. Because a previous study demonstrated that Six1 can have different effects depending on the level of introduced mRNA [34], we also assessed the BOR variants and Six1WT at low (150 pg) and high (400 pg) doses. For *eya2*, both Six1WT-150 and V17E significantly reduced expression; however, there was no significant difference between them indicating V17E was as active as Six1WT. At 400 pg, Six1WT, W122R and Y129C were not significantly different from control or each other, indicating that W122R and Y129C were as active as Six1WT. Interestingly, R110W reduced *eya2* expression significantly more than Six1WT, suggesting that it may be dominant-repressive. For *prdm1*, V17E significantly reduced expression compared to control and Six1WT-150. At 400 pg, R110W and W122R significantly reduced *prdm1* expression below control levels, whereas Six1WT-400 and Y129C were not significantly different from control. For *spry1*, V17E significantly reduced expression below control levels but was indistinguishable from Six1WT-150. At 400 pg, there were no significant differences between controls, Six1-WT and mutants. For *tspan1*, there were no significant differences between controls, Six1WT-150 and V17E; however, at 400 pg R110W significantly reduced *tspan1* expression compared to control and those of Six1WT, W122R and Y129C. For *zbtb16*, there were no significant differences between controls, either level of Six1WT or any of the mutants. For *pa2g4*, Six1WT-150 significantly reduced expression and V17E reduced expression significantly more than Six1WT-150. At 400 pg, Six1WT, R110W and W122R significantly reduced *pa2g4* levels compared to control. Moreover, R110W reduced levels significantly more than Six1WT and Y129C. 

Overall, these results indicate that increased expression of Six1WT significantly reduced the expression in whole heads of some (*eya2*, *pa2g4*) but not all of the measured genes. Importantly, only some of the BOR variants had effects comparable to exogenous Six1WT, indicating that their activity is defective. V17E significantly reduced the expression in whole heads of most genes (*eya2*, *prdm1*, *spry1*, *pa2g4*), sometimes significantly more than a comparable dose of Six1WT (*prdm1*, *pa2g4*). R110W significantly reduced the expression in whole heads of *eya2*, *prdm1*, *tspan13* and *pa2g4*, but this was only significantly different from Six1WT-400 for *tspan13* and *pa2g4*. W122R significantly reduced the expression in whole heads of *prdm1* and *pa2g4*, but they were not significantly different from Six1WT-400. Y129C did not significantly reduce any of the measured genes compared to controls, indicating that is does not have the same activity as Six1WT. Thus, each BOR variant showed differential effects on the cranial expression levels of these genes, which often were different from the effects of exogenously supplied Six1WT. 

### 3.3. BOR Mutations Differentially Affect Otic Gene Expression

**Figure 4:**
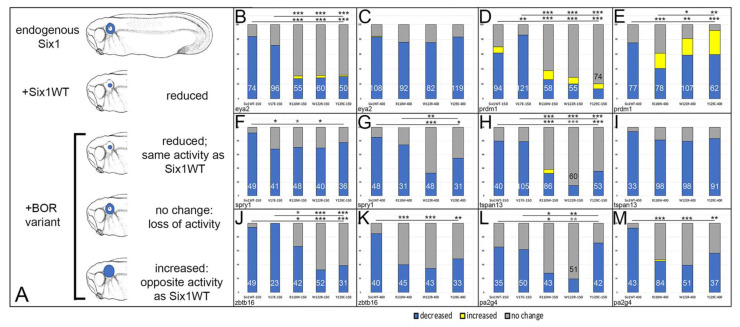
Otic gene expression is altered by Six1 mutants as assessed by ISH. (**A**): Transcripts encoding wild-type Six1 (Six1WT) or BOR variants were microinjected into blastomeres that contribute to otic structures on one side of embryos containing endogenous levels of Six1. The effects on otic vesicle gene expression (blue) were compared between embryos injected with *Six1WT* (+Six1WT) versus those injected with a BOR variant (+BOR variant). Since Six1WT reduced each otic target gene, if the BOR variant also reduced it at the same frequency, then the variant has the same activity as SixWT. If the BOR variant caused no change in expression, then it has lost activity. If the BOR variant increased expression, then it has the opposite activity of SixWT. (**B**). Percentages of embryos in which *eya2* otic expression was decreased (blue), increased (yellow) or not changed (grey) after microinjection of 150 pg of Six1WT, V17E, R110W, W122R or Y129C mRNAs. Numbers inside bars denote number of embryos analyzed. Lower line and asterisks indicate comparison of Six1WT frequencies to those of each mutant. Upper line and asterisks indicate comparison of V17E frequencies to those of the other three mutants. * *p* < 0.01; **, *p* < 0.001; ***, *p* < 0.0001 comparison by chi-square test. (**C**). Percentages of embryos in which *eya2* otic expression was altered after microinjection of 400 pg of Six1WT, R110W, W122R or Y129C mRNAs. There were no significant differences between the different groups. Labeling as in (**B**). (**D**). Percentages of embryos in which *prdm1* otic expression was altered after microinjection of 150 pg of Six1WT, V17E, R110W, W122R or Y129C mRNAs. Labeling as in (**B**). (**E**). Percentages of embryos in which *prdm1* otic expression was altered after microinjection of 400 pg of Six1WT, R110W, W122R or Y129C mRNAs. Labeling as in (**B**). (**F**). Percentages of embryos in which *spry1* otic expression was altered after microinjection of 150 pg of Six1WT, V17E, R110W, W122R or Y129C mRNAs. Labeling as in (**B**). (**G**). Percentages of embryos in which *spry1* otic expression was altered after microinjection of 400 pg of Six1WT, R110W, W122R or Y129C mRNAs. Labeling as in (**B**). (**H**). Percentages of embryos in which *tspan13* otic expression was altered after microinjection of 150 pg of Six1WT, V17E, R110W, W122R or Y129C mRNAs. Labeling as in (**B**). (**I**). Percentages of embryos in which *tspan13* otic expression was altered after microinjection of 400 pg of Six1WT, R110W, W122R or Y129C mRNAs. There were no significant differences between the different groups. Labeling as in (**B**). (**J**). Percentages of embryos in which *zbtb16* otic expression was altered after microinjection of 150 pg of Six1WT, V17E, R110W, W122R or Y129C mRNAs. Labeling as in (**B**). (**K**). Percentages of embryos in which *zbtb16* otic expression was altered after microinjection of 400 pg of Six1WT, R110W, W122R or Y129C mRNAs. Labeling as in (**B**). (**L**). Percentages of embryos in which *pa2g4* otic expression was altered after microinjection of 150 pg of Six1WT, V17E, R110W, W122R or Y129C mRNAs. Labeling as in (**B**). (**M**). Percentages of embryos in which *pa2g4* otic expression was altered after microinjection of 400 pg of Six1WT, R110W, W122R or Y129C mRNAs. Labeling as in (**B**).

BOR phenotypes include tissues that are derived from neural crest (hyoid cysts and fistulas, middle ear, external ear) and the otic placode (inner ear). Because hearing loss is the most consistent BOR phenotype, we sought to determine whether the gene expression differences between wild-type and BOR variants in whole heads (Figure 3) were specifically the result of effects on the inner ear. Therefore, we assessed the expression domains of the Six1 targets in the OV, which gives rise to the entire inner ear. Each BOR variant was expressed in an embryo that contained endogenous levels of Six1, and the frequencies of its effects on OV gene expression were compared to those in embryos injected with Six1WT (Figure 4A). It should be kept in mind that injected mRNAs provide levels of Six1 protein above endogenous Six1. However, this is not expected to result in levels outside normal physiological ranges because (1) Six1 down-regulates its own expression [40], (2) mRNA levels often do not predict protein levels [68], and (3) the mRNA doses used herein previously were shown to differentially affect sox2 expression during cranial ganglion development [34].

At 150 pg, V17E reduced the otic expression of *eya2*, *tspan13*, *zbtb16* and *pa2g4* at a frequency indistinguishable from Six1WT-150 (Figure 4A,G,I,K), whereas it reduced *prdm1* more frequently and *spry1* less frequently compared to Six1WT-150 (Figure 4C,E). At 150 pg, R110W and W122R reduced the otic expression of all six genes at a significantly lower frequency compared to Six1WT-150. At 150 pg, Y129C reduced the otic expression of *eya2*, *prdm1, tspan13*, and *zbtb16* at a significantly lower frequency than Six1WT-150 but showed no difference for *spry1* or *pa2g4*. We also compared the frequencies between the four mutants at 150 pg (Figure 4A,C,E,G,I,K). For *spry1*, the four mutants caused repression at a similar frequency. For *eya2*, *prdm1*, *tspan13* and *zbtb16*, V17E caused significantly more repression of gene expression compared to R110W, W122R and Y129C. For *eya2* and *prdm1*, there were no significant differences between R110W, W122R and Y129C. For *tspan13*, W122R was significantly different from R110W and Y129C was significantly different from W122R. For *zbtb16*, W122R and Y129C were significantly different from R110W. For *pa2g4*, V17E caused significantly more repression compared to R110W and W122R; the effects of V17E and Y129C were indistinguishable. Occasionally, the otic expression of *eya2*, *prdm1* and *tspan13* were broader due to R110W, W122R or Y129C, but this was not observed for V17E.

As previously reported [14], embryos did not survive a microinjection of >150 pg of V17E, whereas R110W, W122R and Y129C were tolerated at a higher concentration. At 400 pg, R110W reduced the otic expression of *eya2*, *spry1* and *tspan13* at the same frequency as Six1WT-400 (Figure 4B,F,H), whereas it reduced *prdm1*, *zbtb16* and *pa2g4* otic expression significantly less frequently than Six1WT-400 (Figure 4D,J,L). W122R and Y129C also reduced the otic expression of *eya2* and *tspan13* at the same frequency as Six1WT-400, whereas they reduced *prdm1*, *spry1*, *zbtb16* and *pa2g4* otic expression significantly less frequently than Six1WT-400. We also compared the frequencies between the three mutants at 400 pg (Figure 4B,D,F,H,J,L). For *eya2*, *tspan13*, *zbtb16* and *pa2g4*, all three mutants reduced gene expression at similar frequencies. For *prdm1*, R110W was significantly less repressive than W122R or Y129C, whereas W122R and Y129C frequencies were indistinguishable. For *spry1*, W122R was significantly less repressive than R110W or Y129C; R110W and Y129C frequencies were indistinguishable. Altogether, these results indicate that the four BOR variants differentially affected the otic expression of Six1 targets. For many of the monitored genes, the mutants were significantly less repressive than Six1WT, indicating loss of activity. In contrast, V17E repressed *prdm1* significantly more than Six1WT, whereas the others caused less frequent reduction and uniquely expanded the expression domain in a subset of embryos (Table 2).

## 4. Discussion

The Six1 homeodomain containing transcription factor plays a key role in vertebrate cranial placode development [15,16,17,18,19]. Loss-of-function by knock-down of endogenous protein or by genetic deletion results in reduced expression of placode genes and a variety of defects during otic development spanning otic placode formation to sensory hair cell differentiation [20,21,22,23,24,25,26,27,28,29,30,31,32,33]. Mutations in human *SIX1* underlie some cases of BOR, but because there is considerable variability in the presence, type and severity of craniofacial and renal abnormalities even among family members carrying the same mutation, it has not been possible to associate a specific mutation with a particular suite of clinical presentations [9,10,11,12,36,37]. One way to begin to address how each mutation might result in a particular phenotype is to study how the variant protein affects developmental processes in an experimental animal model. To this end, we introduced four of the human mutations into *Xenopus* Six1, which has 100% amino acid identity to human SIX1 in the SD and HD. We found that expressing each mutant protein in a wild-type embryo had a different suite of effects on the precursors of tissues affected in BOR, including neural border, neural crest, PPE and a few OV genes [14]. To assess whether these previous observations were the result of Six1 sitting at the apex of the cranial placode gene regulatory network, and thereby altering all downstream events, or the consequence of altering the expression of specific Six1 transcriptional targets, we expanded our analysis to OV genes that previously were reported by large-scale screens to be likely targets of Six1, and we included one novel cofactor [38,39,40,41,42,43]. 

First, we verified that the expression of each of these genes in the OV is downstream of Six1 Using targeted knock-down, we confirmed that Six1 is required for their otic expression (Figure 1; Table 2). Next, we assessed the mode by which Six1 regulates their expression by expressing fusion constructs that render Six1 as either a transcriptional activator (Six1VP16) or a transcriptional repressor (Six1EnR) in the absence of Eya proteins (Figure 2A). Our results indicate that *eya2* and *prdm1* are reduced by exogenously supplied Six1 via transcriptional repression, whereas expression of *spry1*, *tspan13*, and *pa2g4* are reduced by both transcriptional activation and repression (Table 2). In the cases of *tspan13* and *pa2g4*, the mode appears to depend upon the level of Six1 (Table 2), consistent with a previous study that indicated that high levels of Six1 represses *sox2* expression in cranial ganglion precursors, whereas low levels promote it [34]. Although our results cannot discriminate between direct and indirect regulation by Six1, ChIP analysis of the E10.5 mouse OV, which is similar to the OV stage studied herein, identified Six1 binding sites in close proximity to *eya2*, *prdm1*, *spry1* and *zbtb16* genes [42], suggesting that at least some of these effects might be mediated by direct binding to these targets. Other regulators of Six1 transcriptional activity are binding partners. Six1 represses transcription via binding to Groucho/TLE proteins, and activates transcription via binding to Eya proteins, and the latter effect can be attenuated by other proteins in the complex such as Pa2G4 and Mcrs1 [43,69]. Therefore, the mode of Six1 activity reported herein is likely also dependent upon which cofactors, of which there are several candidates [56], are available. A detailed assessment of the components of the Six1 transcriptional complex that sit on identified enhancers of each target gene during OV formation is an important future goal.

Several studies have investigated the biochemical effects of various BOR mutations. For example, a study of *EYA1* mutations concluded that the variant proteins function in a dominant-negative fashion [70]. Based on cell culture experiments, SIX1 mutant proteins have also been suggested to act as dominant-negative by competing with wild-type protein for DNA binding sites or by competing for EYA cofactors [2,71]. With this information in hand, we sought to understand the gene expression phenotypes caused by four of the SIX1 mutants when they are expressed in an embryo containing endogenous levels of Six1. To address whether BOR variants have the same activity in the embryo as exogenously supplied Six1WT, we first assayed gene expression in dissected heads by qPCR. We observed that V17E, R110W and W122R, like exogenous Six1WT, significantly reduced the expression of many of the selected genes, sometimes significantly more than a comparable dose of Six1WT (Figure 3). Interestingly, *zbtb16* expression was not altered by any of the mutants and Y129C did not alter the expression of any of the selected genes. Although these data demonstrate that each SIX1 mutant had a different combination of effects on the cranial expression levels of the target genes, the assay lacks tissue specificity in that whole heads were collected. Therefore, we next expressed the BOR variants only in the blastomere precursors that contribute to the middle and inner ear and assessed OV gene expression by ISH. 

The V17E mutation lies in the first α-helix of the SD. It interferes with the interaction between Six1 and Eya1/Eya2, reduces translocation of cytosolic Eya protein to the nucleus, and is transcriptionally deficient [13,14,71,72]. Our previous work showed that V17E caused the same effects as low levels of Six1WT on neural border gene expression, as well as some NC, PPE and OV genes. However, it reduced the expression of *sox9* in the neural crest and otic placode less than Six1WT. In contrast, it reduced *irx1* in the PPE more than Six1WT, reduced *sox11* in the PPE, whereas Six1WT expanded *sox11* and had differential effects on OV genes. *pax2* was reduced less by V17E than by Six1WT, whereas *sox9*, *tbx1* and *dlx5* were reduced more than by Six1WT, and V17E reduction of *irx1* and *otx2* expression was similar to Six1WT [14]. Of these otic genes, there is only evidence for *irx1* to be a direct target of Six1 [33,42], so we investigated the effects on otic genes that are likely to be targets. Similar to Shah et al. [14], V17E reduced one otic gene less (*spry1*), and one otic gene (*prdm1*) more, compared to Six1WT, but most effects were indistinguishable from Six1WT (Table 2). Since increased Six1 protein predominantly acts as a transcriptional repressor of these genes (Figure 2; Table 2), we propose that for *eya2*, *tspan13*, *zbtb16* and *pa2g4*, there is sufficient endogenous wild-type Six1 to drive their expression even in the presence of V17E. Since binding to Eya has been proposed to stabilize Six1 [13,72], the hypomorphic effect of V17E on *spry1* may be due to protein degradation. The dominant-negative effect of V17E on *prdm1* might be due to competition with wild-type Six1 for DNA binding sites, as suggested for Eya1 BOR variants [70]. 

The R110W mutation lies in the sixth α-helix of the SD. It reduces the interaction with EYA1 but not with EYA2 [10,13]. It can transport EYA2 and Eya1 to the nucleus [13,14] and bind to DNA [10,13], but does not activate transcription in a luciferase reporter assay in the presence of Eya1 or EYA2 [10,13,14]. Our previous work showed that R110W reduced neural border, NC and PPE gene expression significantly less than Six1WT, and sometimes caused them to be broader. R110W (400 pg) reduced the otic expression of *pax2*, *sox9*, *tbx1* and *dlx5* significantly less than Six1WT (400 pg), whereas the reduction of *irx1* and *otx2* expression was similar to that of Six1WT [14]. In the present study, we assessed the effects of both a low dose (150 pg) and high dose (400 pg) of Six1WT and BOR variants. At 150 pg, R110W reduced each otic target significantly less frequently than Six1WT, and at 400 pg it reduced *prdm1*, *zbtb16* and *pa2g4* significantly less frequently. Together, these studies indicate that the R110W variant acts as a hypomorph by rendering Six1 less effective at repressing target genes. Since R110W can bind cytosolic Eya1 and transport it to the nucleus and bind DNA, we posit that this loss of activity may be due to interference with the binding of cofactors that mediate transcriptional repression, such as Groucho/TLE, Pa2G4 and Mcrs1 [43,56,69]. It is interesting that at high levels of expression, it can have either the same activity as exogenous Six1WT or the opposite activity, depending on the target gene. These latter effects might be explained by binding to low affinity sites in the presence of excess protein, as has been reported for numerous transcription factors [73].

The W122R mutation, located in the linker region between the sixth α-helix and the HD, has been postulated to affect DNA binding efficiency [13]. Although it can translocate Eya1 to the nucleus in HEK293T cells, it cannot activate transcription in the presence of Eya1 [14]. Our previous work showed that W122R reduced neural border, NC and PPE gene expression significantly less than Six1WT, and sometimes caused them to be broader. W122R (400 pg) reduced the otic expression of *pax2* and *sox9* significantly less than Six1WT (400 pg), whereas the reduction of *tbx1*, *dlx5*, *irx1* and *otx2* expression were similar to that of Six1WT [14]. In the present study, at 150 pg W122R reduced all 6 six otic genes significantly less frequently than Six1WT, indicating it is hypomorphic and has lost activity. At 400 pg, it similarly reduced *prdm1*, *spry1*, *zbtb16* and *pa2g4* significantly less frequently, but had activity similar to Six1WT for *eya2* and *tspan13*, and the opposite activity for *prdm1*. Since these results are very similar to those of R110W, we predict that its effects are due to deficits in binding repressive cofactors rather than deficits in DNA binding.

The Y129C mutation, located in the N-terminal region of the HD, can interact with Eya1/EYA2 and translocate them to the nucleus, but it does not bind to DNA or activate transcription in a luciferase reporter assay in the presence of Eya1 or EYA2 [10,13,14]. Our previous work showed that Y129C caused the same effect as Six1WT on neural border gene expression [14], but it reduced NC and PPE gene expression significantly less than Six1WT, and sometimes caused them to be broader. Y129C (400 pg) reduced the otic expression of *pax2* and *sox9* significantly less than Six1WT (400 pg), whereas the reduction of *tbx1*, *dlx5*, *irx1* and *otx2* expression was similar to that of Six1WT [14]. In the present study, at 150 pg Y129C reduced the otic expression of *eya2*, *prdm1*, *tspan13* and *zbtb16* significantly less frequently than Six1WT, indicating it is hypomorphic and has lost activity. At 400 pg, it reduced *prdm1*, *spry1*, *zbtb16* and *pa2g4* significantly less frequently. If Y129C acted as a dominant-negative by reducing the available endogenous Eya1, as previously postulated [2,72], we predict it would cause endogenous Six1WT to bind to target genes without Eya1 and thus be more repressive; we did not observe this to be the case. Accordingly, perhaps Y129C, as proposed for W122R, binds to and thereby reduces the endogenous availability of repressive cofactors.

### Do the Effects of BOR Variants Correlate with Patient Phenotypes?

We chose to study the effects of two BOR variants that are found in the SD (V17E, R110W), and thus are likely to interfere with cofactor binding, and two that are adjacent to or within the HD (W122R, Y129C), and thus are likely to interfere with DNA binding. Because the most commonly affected craniofacial structures i.e., hyoid arch, inner, middle and external ears, are derived from the cranial neural crest and placodes, we predicted that the BOR variants would alter gene expression in these precursor populations in the early embryo. Indeed, this was observed [14], but to our surprise, each variant caused a different but overlapping suite of effects on the various genes we monitored, which did not correlate with whether the variant was in the SD or HD and did not correlate in any detectable way with reported patient phenotypes. Thus, we decided to focus on the one tissue that is consistently affected, i.e., the OV precursor of the inner ear (Table 2), and to examine only genes that are likely to be regulated, either directly or indirectly, by Six1. As reported with neural crest and placode genes [14], we found that each BOR variant rarely affected otic target genes in a consistent manner, indicating that both direct and indirect effects were in play. We also found that each mutant tended to show a lower frequency of reduced gene expression compared to Six1WT, indicating loss of activity (Table 2). Since Six1 acts as a transcriptional repressor in the absence of Eya1, and given that R110W, W122R and Y129C can bind Eya1 and transport it to the nucleus, we propose that one likely cause of these mutants showing a less reduced phenotype is that they do not bind to repressive cofactors, such as Groucho/TLE, Pa2G4 or Mcrs1. Alternatively, altered *pa2g4* expression may account for these changes, since Pa2G4 is a repressive cofactor that competes with Eya1 binding [43]; the lower frequency of reduced *pa2g4* would result in higher levels of its protein being available. It is interesting that V17E tends to have effects that are significantly different from R110W, W122R and Y129C, suggesting that the underlying cause is its inability to bind cytosolic Eya proteins and translocate them to the nucleus. Although it has been suggested that V17E protein is less stable because it does not bind Eya proteins [13], in most of our assays it had activity similar to Six1WT. An important next step in understanding these changes in gene expression is to identify the function of each of the several candidate cofactors [55], to identify whether their binding sites in the SD coincide with known BOR mutations, and whether they interfere with Eya protein binding.

## Figures and Tables

**Table 1:** qPCR primer sequences.

Gene	Forward Sequence	Reverse Sequence
eya2	CCTCGGACGACAATGGACAA	CAGTCAACTCCCCCATGGAC
prdm1	AAGGAACACGGTTTGGACCA	TGAAGTGCTGGAAGTCACCA
spry1	TGCTTGCACAGAGGTTTTCAG	TTGTAGCTCCATCTGTAGTGATCT
tspan13	CATGCGCGTCTCTGGCTATA	AGCCCCACAGGTGTCATTTT
zbtb16	GGGTGTGAGCTCTGTGGAAA	ACACACAAATGCCTTTGCCC
pa2g4	GCCTGAAAATGAAAACCTCCC	TTCCACAACTCCCATTCTCG

**Table 2:** Summary of effects Six1 on otic vesicle gene expression.

Gene	Is Six1 Required?	Does Six1 Activate or Repress?	V17E	R110W	W122R	Y129C
eya2 *	yes	repress	same as Six1WT	low: loss of activity high: same as Six1WT	low: loss of activity high: same as Six1WT	low: loss of activity high: same as Six1WT
prdm1 *	yes	repress	more repressive than Six1WT	low: loss of activity high: loss of activity & opposite activity	low: loss of activity high: loss of activity & opposite activity	low: loss of activity high: loss of activity & opposite activity
spry1 *	yes	both	loss of activity	low: loss of activity high: same as Six1WT	low: loss of activity high: loss of activity	low: same as Six1WT high: loss of activity
tspan13	yes	low: repress high: activate	same as Six1WT	low: loss of activity high: same as Six1WT	low: loss of activity high: same as Six1WT	low: loss of activity high: same as Six1WT
zbtb16 *	yes	---	same as Six1WT	low: loss of activity high: loss of activity	low: loss of activity high: loss of activity	low: loss of activity high: loss of activity
pa2g4	yes	low: activate high: repress	same as Six1WT	low: loss of activity high: loss of activity	low: loss of activity high: loss of activity	low: same as Six1WT high: loss of activity
Patients			hearing loss hyoid fistulae preauricular pits [12]	hearing loss hyoid cysts (v) preauricular pits (v) pinnae defect (v) renal (v) [10,12]	hearing loss hyoid fistulae (v) preauricular pits (v) [11]	hearing loss hyoid fistulae (v) preauricular pits (v) [9]

Legend: * evidence that Six1 binds to nearby enhancer in mouse E10.5 otic vesicle [42]; “same as Six1WT” = reduced as frequently compared to embryos injected with Six1WT; “loss of activity” = reduced significantly less frequently compared to embryos injected with Six1WT; “more repressive than Six1WT” = reduced significantly more frequently compared to embryos injected with Six1WT;“opposite activity” = expanded in many embryos, whereas Six1WT always caused reduction; ---, data reported in a pending, unpublished manuscript; (v), variable phenotype even within a family.

## Data Availability

Not applicable.
